# IgA Vasculitis With Adalimumab Therapy for Hidradenitis Suppurativa: A Case Report and Review of Literature

**DOI:** 10.7759/cureus.30122

**Published:** 2022-10-10

**Authors:** Sarah Alsulami, Rakan M Alotaibi, Faisal F Alotaibi, Faisal B Almatrafi, Doaa M Filmban, Sultan Alneefia, AhmedHusam Alahmed, Waleed A Hafiz

**Affiliations:** 1 Department of Medicine, Al Noor Specialist Hospital, Makkah, SAU; 2 Department of Medicine, College of Medicine, Umm Al-Qura University, Makkah, SAU

**Keywords:** hidradenitis suppurativa, rheumatology, dermatology, anti-tnf agents, vasculitis, anti-tnf alpha, henoch schönlein purpura, adalimumab, adult iga vasculitis

## Abstract

Immunoglobulin A (IgA) vasculitis is a systemic vasculitis characterized by inflammation of the small vessels, with cutaneous, musculoskeletal, gastrointestinal, and renal involvement, usually seen in pediatric populations. Hidradenitis suppurativa is a chronic inflammatory disorder of the skin, which can be treated by tumor necrosis factor-α (TNFα) inhibitor therapy. TNFα inhibitor therapy is used as an important milestone in the treatment of various rheumatological and autoimmune disorders. Unexpected adverse effects might occur. However, they are usually mild and do not warrant treatment withdrawal. We present a case of IgA vasculitis complicating adalimumab therapy for hidradenitis suppurativa. We also review and discuss similar cases reported in the literature.

## Introduction

Immunoglobulin A (IgA) vasculitis, formerly Henoch-Schönlein purpura, is a systemic immune complex vasculitis primarily affecting the small vessels [1]. IgA vasculitis is the most common systemic vasculitis in the pediatric population. Its annual incidence is three to 26 cases per 100,000 children [2]. It is more common in winter and fall and in pediatric patients between the ages of four and seven years. The condition is less common in adults, with an annual incidence of 0.1 to 1.8 per 100,000 adults, and is noted to occur more frequently in summer and winter [3,4].

Until now, the etiology of IgA vasculitis remains unknown. However, the deposition of IgA1-dominant immune complexes in target organs plays a pivotal role in the pathophysiology of the condition [5,6].

The clinical scope of the disorder essentially includes cutaneous purpura, arthralgias and/or arthritis, glomerulonephritis, and acute enteritis [4]. These clinical presentations may develop within days to weeks and differ in their sequence of manifestation. Palpable purpura has been reported in 96-100% of cases at presentation, arthralgia and/or arthritis are seen in 61%, and gastrointestinal features in 48-53% of the cases. Approximately one-third of the cases develop renal failure with a creatinine clearance of <50 ml/min/m² within four months of presentation [4,7].

The American College of Rheumatology 1990 criteria for the classification of IgA vasculitis are utilized for diagnostic purposes. These criteria include the following: age at onset of 20 years or more, the presence of a slightly elevated palpable purpuric rash, acute abdominal pain, and biopsy showing granulocyte in the walls of small arteries. The presence of at least two criteria carries a sensitivity of 87.1% and a specificity of 87.7% for the diagnosis of IgA vasculitis [8].

Hidradenitis suppurativa (HS) is a chronic inflammatory, recurrent, and debilitating skin disorder that usually presents after puberty with painful, deeply located, and inflamed lesions in the apocrine gland-bearing tissues, usually in the axillaries, inguinal, and anogenital areas [9]. It affects 1% of the global population [10].

HS significantly decreases the quality of life. Patients report self-consciousness, embarrassment, and inability to participate in athletic and social activities [11].

This report presents a case of IgA vasculitis in a patient receiving adalimumab for HS.

## Case presentation

A 26-year-old man presented to the emergency department with moderate diffuse abdominal pain and painless purpuric erythematous rash over his lower extremities. It was associated with bilateral lower limb swelling and pain with swelling and stiffness of both ankles and left knee. His symptoms began two weeks ago and progressed gradually. He was clinically assessed in a private clinic and was treated with prednisone 30 mg daily for a short period and a quick taper. His symptoms responded marginally to prednisone for a few days. Then, his abdominal pain became severe with associated nausea, non-projectile and non-bloody vomiting, and a single episode of bloody diarrhea. His rash further extended to involve his upper thigh and buttock area. He denied any constitutional symptoms, headache, dizziness, chest pain, palpitation, shortness of breath, or change in his urine color.

His past medical history is significant for hidradenitis suppurativa for three years, for which he follows up with dermatology and has been on adalimumab 80 mg every other week for two years. He is not on any other medications and is allergic to penicillin and sodium diclofenac. There was no history of recent travel, contact with sick patients, or infection. He has no family history of autoimmune diseases, malignancies, or similar conditions. Past surgical history showed gastric sleeve surgery three years ago with no complications.

**Figure 1:**
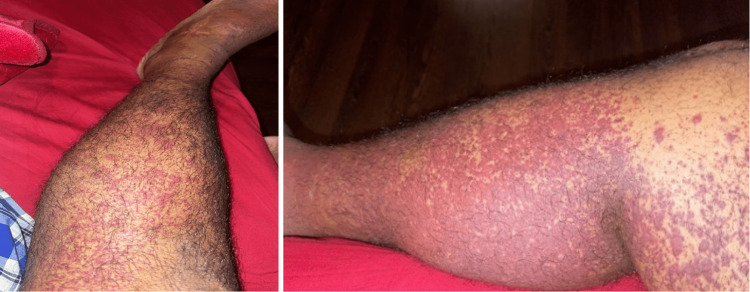
Classic palpable purple-colored purpura involving the lower extremities of the patient.

On physical examination, he looked in pain but not in distress. His weight is 72 kg, with a height of 168 cm and a body mass index of 25.5. His vital signs were all normal. His abdomen was soft and lax, with only moderate epigastric tenderness and no organomegaly or guarding. Lower extremity examination revealed diffuse non-tender raised purpuric rash over both shins and thighs with no evidence of bleeding or discharge (Figure 1). Chest, cardiovascular and neurological examination was unremarkable.

The results of the laboratory investigation are listed in Table 1. Urinalysis did not show any evidence of hematuria. Abdominal ultrasound showed normal liver, gall bladder, spleen, and kidneys.

**Table 1:** Laboratory investigations for the patient were done upon his presentation to the emergency department.

Test	Result	Normal range	Unit
Hemoglobin	138	130-180	g/L
White blood count	7.72	4-11	10^9^/L
Platelet count	297	150-400	10^9^/L
Prothrombin time	13.4	11-16	Second
Partial thromboplastin time	27.8	26-36	Second
International normalized ratio	1.17	0.8-1.2	
Blood glucose	6.2	3.6-6.1	mmol/L
Blood urea nitrogen	3	3.2-8.2	mmol/L
Creatinine	51	62-115	umol/L
Sodium	132	135-145	mmol/L
Potassium	3.4	3.5-5.1	mmol/L
Total bilirubin	16	0-18.7	umol/L
Direct bilirubin	6	0-5	umol/L
Alanine aminotransferase	10	10-49	U/L
Aspartate aminotransferase	16	10-34	U/L
24-hour urine for protein	137.7	0-150	Mg/day

Based on that, he was admitted to the medical ward as a case of IgA vasculitis associated with hidradenitis suppurativa under adalimumab therapy. Adalimumab was stopped, and the patient was treated with intravenous pulse methylprednisolone at a dose of 500 mg once daily for three days and supportive therapy. After three days of admission, he responded dramatically to this treatment regimen with significant improvement in his abdominal pain, arthritis, and skin rash. For that, he was discharged home after three days of inpatient management to continue on oral prednisone 60 mg once daily for two weeks with a quick taper to nil over one month with rheumatology follow-up since September 2021 until now.

## Discussion

Tumor necrosis factor-α (TNFα) is a pleiotropic cytokine that is known to play a role in host defense mechanisms and initiates the response to local injury [12]. Currently, there are multiple anti-TNFα, which are monoclonal antibodies, one of which is adalimumab [13]. Many side effects are associated with anti-TNFα therapy, and they include increased risk of infections such as tuberculosis, malignancies, infusion- or injection-related skin reactions, cardiovascular disease, and autoimmunity [14].

It is uncommon for adalimumab to be involved in association with IgA vasculitis. However, a review of the literature revealed two cases of IgA vasculitis in patients under adalimumab therapy.

The first case is a 19-year-old male, known case of Crohn's disease on adalimumab, who presented with a 10-day history of diffuse arthritis and a non-blanchable purpuric rash on both legs that were preceded by a short-term coryzal illness. Initially, the patient was managed conservatively, and a multi-disciplinary decision was made to commence a trial of adalimumab. However, the patient returned three days later with recurrence and an inability to bear weight in the lower extremity joints. The patient was then treated with right ankle intra-articular steroids along with systemic steroids as adalimumab was discontinued. His IgA vasculitis-related symptoms resolved entirely after a few weeks [15].

The second case is a 33-year-old Caucasian man known to have ulcerative colitis for three years on adalimumab who was admitted to the hospital for a recurrent erythematous, palpable, non-blanching purpuric rash in the lower extremities bilaterally with arthritis in the ankles, knees, and elbows. The rash recurred with more severity and ascended to the buttocks, lower back, and abdomen. Adalimumab was stopped, and the patient was treated with methylprednisolone 20 mg intravenously every eight hours. As a result, the rash and arthritis improved significantly [16].

Table 2 provides a summary of all cases of IgA vasculitis associated with adalimumab therapy that are reported in the literature.

**Table 2:** Summary of our case and similar reported cases in the literature.

	Our case	The first case by Rahman et al. [15]	The second case by LaConti et al. [16]
Clinical data	A 26-year-old male known case of hidradenitis suppurativa on adalimumab presented with moderate to diffuse abdominal pain with an erythematous painless rash over his lower extremities, with bilateral swelling and stiffness of both ankles and left knee along with diffuse purpuric rash in the buttocks and upper thigh.	A 19-year-old male known case of Crohn's disease on adalimumab presented with a 10-day history of diffuse arthritis in both lower extremities, both hands, and right elbow joints associated with non-blanching purpuric rash in the lower limbs.	A 33-year-old Caucasian male with ulcerative colitis on adalimumab presented for work of recurrent erythematous, palpable, non-blanching rash in his bilateral lower extremity, buttocks, lower back, and abdomen, associated with joint pain and swelling of his ankles, knees, and elbows.
Management	He was admitted to the medical ward as a case of IgA vasculitis. He was treated with intravenous pulse methylprednisolone 500 mg daily for three days, with supportive management. Adalimumab was discontinued. He was discharged after three days with a rheumatology follow-up.	At first, the patient was treated conservatively, and adalimumab was resumed initially. However, his symptoms worsened three days after discharge, and was started on right ankle intraarticular steroids and systemic steroids, as adalimumab was stopped.	The patient received treatment with methylprednisolone 20 mg intravenously every eight hours for one week as adalimumab was stopped with follow-up.
Outcome	All IgA vasculitis-related symptoms improved dramatically after three days of admission.	After a few weeks, his IgA vasculitis-related symptoms resolved completely.	The rash almost completely resolved.

HS has been reported to be associated with multiple types of vasculitides. A case series and literature review revealed a rare association between HS and vasculitis seen in five reported cases. Three out of these showed an association between granulomatosis with polyangiitis and HS. Additionally, one reported case demonstrated an association between Behcet's disease and HS. Finally, one reported case showed an association between Takayasu's arteritis and HS [17].

According to our knowledge, our case is the first that may demonstrate an association between IgA vasculitis and adalimumab as a therapy for a co-associated HS. Additionally, our case adds to the observation of an increasing number of IgA vasculitis cases in association with adalimumab therapy. Based on the literature earlier, HS and/or adalimumab may be both contributing risk factors to the onset of IgA vasculitis through an unknown mechanism.

## Conclusions

There is a rare association between IgA vasculitis and anti-TNFα biological agents. This limited but seemingly growing evidence in the literature suggests that anti-TNFα may elicit the development of IgA vasculitis. We support this theory suggested by Rahman et al. and LaConti et al. Concurrently, literature also suggested that HS might promote vasculitis. We believe further studies are needed to determine the pathophysiological link and predisposition in the inhibition of TNFα and the presence of HS that lead to the deposition of IgA complexes systemically.
